# Exploring the Interaction Effects of Gender Contentedness and Pubertal Timing on Adolescent Longitudinal Psychological and Behavioral Health Outcomes

**DOI:** 10.3389/fpsyt.2021.660746

**Published:** 2021-11-26

**Authors:** Jen-Hao Kuo, Raúl Albaladejo Carrera, Lidya Cendra Mulyani, Carol Strong, Yi-Ching Lin, Yi-Ping Hsieh, Meng-Che Tsai, Chung-Ying Lin

**Affiliations:** ^1^School of Medicine, College of Medicine, National Cheng Kung University, Tainan, Taiwan; ^2^Faculty of Medicine and Odontology, University of Valencia, Valencia, Spain; ^3^Faculty of Medicine, University of Pelita Harapan, Tangerang City, Indonesia; ^4^Department of Public Health, National Cheng Kung University Hospital, College of Medicine, National Cheng Kung University, Tainan, Taiwan; ^5^Department of Early Childhood and Family Education, College of Education, National Taipei University of Education, Taipei, Taiwan; ^6^Department of Social Work, College of Nursing and Professional Disciplines, University of North Dakota, Grand Forks, ND, United States; ^7^Department of Pediatrics, National Cheng Kung University Hospital, College of Medicine, National Cheng Kung University, Tainan, Taiwan; ^8^Institute of Allied Health Sciences, College of Medicine, National Cheng Kung University, Tainan, Taiwan

**Keywords:** adolescent, gender contentedness, pubertal timing, depression, behavioral development

## Abstract

**Background:** Off-time pubertal timing (PT) and non-conforming gender identity have been reported to predict adverse health and well-being in adolescents. However, the joint effects of these two factors are less addressed. We aimed to investigate the main and interaction effects of gender identity, proxied by perceived gender contentedness (GC), and PT on longitudinal adolescent psychological and behavioral outcomes.

**Methods:** Data (*N* = 1806, M*age* = 13.3 ± 0.5 years) come from the Taiwan Youth Project, which prospectively followed a longitudinal cohort of Taiwanese junior high school students from 2000 (wave 1) to 2009 (wave 9). GC was self-reported at waves 1 and 9 in a binary response, and thus 4 GC trajectories were created. PT was defined using the Pubertal Developmental Scale, which mainly measured physical changes in puberty. Multiple linear regression analyses with gender stratification were applied to examine the effects of the GC trajectory and its interaction with PT on the outcomes.

**Results:** A total of 1,562 subjects (86.5%) remained consistently satisfied with their gender, while the GC of 226 subjects (12.6%) changed at some point. Regression analyses found that males with gender dissatisfaction at wave 9 were likely to engage in delinquent behavior, and females in this group were more likely to have lower self-esteem, as compared to those with consistent GC. The interaction effect between the GC trajectory and PT appeared to be associated with smoking and drinking only at wave 1.

**Conclusions:** These findings indicate that healthcare professionals should concentrate on gender non-conforming individuals at early adolescence, navigating them toward a healthy adulthood.

## Introduction

Gender identity usually refers to the extent to which a person adheres to one gender, which in turn determines self-image and provides an important basis for social interaction (1). It has been widely observed that gender conformity is linked to psychological well-being in adolescents and young adults (2). Gender non-conforming children and adolescents are likely to have mal-adjustment issues and thus to be at risk for emotional disturbance such as anxiety, depression, and even suicidal ideations (3, 4). Especially during adolescence, gender identity develops in sequential steps through an intertwined process of personal reflection and contextual influence. For example, increased pressure from family and school may force adolescents to conform to culturally sanctioned gender roles, which results in a further differentiation in gender-role identification in boys and girls (5). Prepubertal issues of gender identity typically resolve before adolescence but they may persist lifelong once past puberty (6). How the temporal trajectory of changes in gender identity is related to developmental outcomes, which has been rarely addressed in cross-sectional studies, may require further research.

Egan and Perry proposed a multidimensional model of gender identity measured using an instrument that includes individuals' perceptions of how typical they are for their gender (gender typicality), how content they are with their socially prescribed gender role (gender contentedness, GC), and how pressured they feel to conform to gender norms (experience pressure from peers and parents) (7). As argued, gender-atypical behaviors and dissatisfaction with one's gender assignment may co-occur, which may suggest that self-perceived gender atypicality and gender discontentment are two correlated indexes of a common and more global underlying factor of felt gender compatibility (8). For a developing child and adolescent, GC may therefore be a reliable alternative indicator of gender identity/typicality, a less pathologized social construction before the perception of gender identity is completely concrete at a later age. Therefore, we explored the issue of gender identity using the proxy of GC in this paper. Moreover, some gender development theories have defied the stability of gender-typed distinction across development in childhood to adolescence (9). For instance, based on cognitive developmental theory, Carol Martin and her colleague found that development of gender identity in early adolescence would change owing to maturation of cognitive ability and evolving understanding of gender concepts (10). Nonetheless, it remained to be tackled whether and when children and adolescents change gender identify with age.

Pubertal timing (PT) is another critical factor that has been shown to be associated with adolescent development and to predict health status in young adulthood (6, 11). Substantial evidence has demonstrated a higher likelihood of having depressive symptoms, exhibiting delinquent behavior, substance abuse, and risky sex among early maturing individuals (12–14). The effects of PT on adolescent development are consistently manifested until young adulthood (15, 16). There is some evidence showing a significant link between sexual orientation and age of pubertal onset (17), which is less so in the relationship with gender identity. However, studies on gender dysphoria and disorders of sex development highlight the period of adolescence as a critical transition for the development of gender non-conformity due to the action of pubertal hormones and the changing social environment (1). For example, the onset of physical changes at puberty may worsen symptoms of gender dysphoria and increase destructive behavior in gender non-conforming adolescents (2). Given these issues, little research has been dedicated to exploring potential interaction effects between GC and PT on adolescent development. One earlier cross-sectional study conducted on Hong Kong adolescents found that GC was no longer a significant contributor to depressive symptoms when the other predictors, such as early PT and intercourse, were included (18). However, this finding has not been replicated in a longitudinal research design, and whether it is applicable to other behavioral outcomes remains unclear.

In Taiwan, there were 7.3% in females and 1.9% in males self-reporting gender dysphoria according to a questionnaire-based screening among incoming university students (19). Further exploring the correlates of gender dysphoria in this age group, results found that gender non-conforming students had more co-morbid psychiatric symptoms, such as anxiety, depression, body dysmorphia, and suicidal ideation (19, 20). However, there has been no published survey on gender issues in Taiwanese adolescents, and how this issue is related to individual psychological and behavioral outcomes remains unclear. In this study, we sought to investigate the longitudinal effects of GC and its interaction with PT on youth psychosocial and behavioral outcomes. Using the data from a prospectively followed cohort, we were able to capture the changes in GC over the entire adolescent period so that the GC trajectory was the main predictive variable of interest in the analysis while the role of PT was jointly considered. We hypothesized that the GC trajectory significantly impacts youth development and that PT may moderates this impact.

## Methods

### Study Population

The data used in this study were retrieved from the Taiwan Youth Project (TYP), which was launched by the Institute of Sociology, Academia Sinica, Taiwan (21). In brief, junior high school students were recruited in 2,000 and followed up annually. The participants were recruited from northern Taiwan, including Taipei City, New Taipei City (called Taipei County before 2010), and Yilan County, using a multistage-stratified and class-clustered selection procedure. A subset of data (*N* = 1,806, M*age* = 13.3 ± 0.5 years) on 7th graders collected items related to behavioral and psychological health from 2000 (wave 1) to 2009 (wave 9), where they were 22.3 ± 0.5 years, and thus were deemed valid for the analysis. The study was approved by the Institutional Review Board of the National Cheng Kung University Hospital.

### Measures

#### Gender Contentedness

Considering the availability of data in the TYP, GC was originally assessed using a single item “Are you satisfied with your own gender?” rated on a 4-point Likert scale from 1 (very dissatisfied) to 4 (very satisfied), which we aligned with the literal meaning of the answers and regrouped them into binary items, i.e., satisfied and dissatisfied, to indicate overall felt gender compatibility (7, 22).

Because the measurement of GC was only available at waves 1 and 9, we created 4 coherent GC trajectory groups based on the values at these two waves. GC1 represented those who were consistently satisfied with their gender at both waves. GC2 referred to those who were not satisfied with their gender at wave 1 but became satisfied at wave 9. GC3 was defined as those who were satisfied with their gender at wave 1 but not satisfied at wave 9. GC4 was defined as those who were consistently dissatisfied with their gender at both waves.

#### Pubertal Timing

PT was assessed based on the items of the Pubertal Developmental Scale (PDS), which measured self-perceived physical changes, including height spurts, body hair development, skin changes, breast growth/deepening of voice, and menarche/facial hair development. Except for menarche, which was a dichotomous item (“yes” or “no”), all other items were rated using a 4-point Likert scale. Aligned with prior research, we summarized the PDS scores and standardized them within same-sex and same-age cohorts (in years) to represent PT among the participants (22, 23), where a higher score represented earlier PT.

#### Outcome Variables

Delinquent behavior that participants were asked to report on for the previous year included “vandalism,” “stealing,” “cheating,” and “using illicit drugs.” These four items were chosen because they represented the most common deviant behaviors among Taiwanese adolescents and were available throughout all waves in the TYP dataset (21). Responses were rated on a 5-point Likert scale that corresponded to the frequency of each item from “never” to “always.” For analytic purposes, we added them to create a single scale (Cronbach α = 0.674) that indicated the degree of behavioral problems, with a higher score representing more delinquent behaviors.

Psychological well-being was measured on 16 major physical and psychological symptoms of the Center for Epidemiologic Studies Depression (CES-D) scale. It has been supported that CES-D was a useful tool to understand the concept of depression in Taiwanese adolescents. It typically represent adolescent mental health-related investigations, including “headaches,” “dizziness,” “loneliness,” “depression,” “worriedness,” “feeling like hurting others,” “feeling like arguing with others,” “feeling like screaming,” “insomnia,” “waking up early,” “light sleeping,” “muscle pain,” “feeling numb,” “feeling like something is stuck in your throat,” “feeling weak,” and “having suicidal feelings” (24). This scale has been extensively applied in research on depression and psychological well-being in this local setting (25–27). A 5-point Likert scale was applied to rate these items, and all the item scores were added together to create a score representing depressive symptomatology (Cronbach α = 0.844). The higher the score, the poorer psychological well-being the respondent was considered to have.

Self-esteem was measured using 9 items of the Rosenberg Self-Esteem Scale on a 4-point Likert scale, ranging from 1 (strongly agree) to 4 (strongly disagree) (Cronbach α = 0.685). A higher score on this scale represented higher self-esteem (28).

Alcohol drinking and cigarette smoking were self-reported by the participants according to the frequency of use. Although the two behaviors are considered illegal and inappropriate for teenagers (wave 1), they are generally unhealthy but accepted social activities in young adulthood (wave 9). Thus, we separated the two items from the other delinquent behaviors in the analysis. The answers were categorized into five brackets, where “none,” “1–2,” “3–4,” “5–6,” and “more than 6 times/month” represented alcohol drinking within 1 month, and “none,” “ <1 pack per week,” “one pack per week,” “two packs per week,” and “more than two packs per week” represented cigarette smoking within 1 week (22, 29). For analytic purposes, the frequency of alcohol and cigarette use was scored from 1 to 5 separately and then added to create a single score representing the use of these two substances.

#### Covariates

Socioeconomic covariates included parental education and family incomes. The parent with the most education of the two parents was used as the reference for parental education. Monthly family income was subdivided into three groups: “New Taiwanese dollar (NTD) 30,000 or less,” “NTD 30,001–60000,” and “NTD 60,001 or more.”

#### Statistical Analysis

All the analyses were conducted using SPSS15.0 (SPSS Inc., Chicago, IL). The baseline socio-demographic data were compared, after gender stratification, between the different GC trajectory groups using a one-way analysis of variance (ANOVA) with a Bonferroni adjustment to identify significant differences in multiple comparisons. In order to examine the effects of the GC trajectory and PT on the outcome variables, we further used multiple linear regression analyses, where gender was stratified, and the covariates (parental education and family income) were adjusted. Specifically, Model 1 tested the sole effect of the GC trajectory, and Model 2 tested the concurrent effects of the GC trajectory and PT on the outcome variables at waves 1 and 9, respectively. When analyzing the outcome variable at wave 9, we also included the value at wave 1 in the model to adjust for its time-dependent effect. In Model 3, we further added the interaction term between the GC trajectory and PT in the full model. Because of a limited number in GC3 and GC4 groups, we combined these two groups into one (GC3+4), which represented those who were not satisfied with their gender at wave 9, in these exploratory analyses. GC1 was used as the reference group in all of the analyses, and a coefficient (β) along with a 95% confidence interval (CI) was given to describe the estimated effects for the other GC trajectory groups.

## Results

Among the 1,806 participants, 1,562 (86.5%) were consistently satisfied with their gender; 226 (12.6%) changed at some point between waves 1 and 9, and 18 (0.9%) were consistently dissatisfied (Table 1). When comparing the outcome variables among the different GC trajectory groups, we found that boys in GC2 had poorer psychological well-being and lower self-esteem than those in the GC1 and GC3 groups at wave 1 (*F* = 5.31, *p* < 0.01 and *F* = 6.92, *p* < 0.01, respectively) (Table 2). Meanwhile, boys in GC3 were found to engage in more delinquent behaviors than those in the GC1 and GC2 groups at wave 9 (*F* = 4.26, *p* < 0.01). In their female counterparts, those in GC4 had the poorest psychological well-being (*F* = 15.3, *p* < 0.01) at wave 1 and the highest risk for delinquent behavior (*F* = 5.9, *p* < 0.01) and use of cigarettes and alcohol (*F* = 6.04, *p* < 0.01) at wave 9 (Table 2).

**Table 1:** Demographic information of participants (*N* = 1,806).

	**All, *n* (%)**	**Male, *n* (%)**	**Female, *n* (%)**
GC trajectory groups	1,806 (100.0)	921 (100.0)	885 (100.0)
GC1	1,562 (86.5)	844 (91.6)	718 (81.1)
GC2	140 (7.8)	30 (3.3)	110 (12.4)
GC3	86 (4.8)	44 (4.8)	42 (4.7)
GC4	18 (0.9)	3 (0.3)	15 (1.7)
Pubertal timing, mean (s.d.)	8.82 (2.22)	9.53 (2.40)	8.09 (1.73)
Monthly family income (NTD)			
≦30,000	282 (15.6)	134 (14.5)	148 (16.7)
30,001–60,000	719 (39.8)	358 (38.9)	361 (40.8)
≧60,001	640 (35.4)	351 (38.1)	289 (32.7)
Missing	165 (9.1)	78 (8.5)	87 (9.8)
Parents' education			
Junior high school or lower	582 (32.2)	295 (32.0)	287 (32.4)
Senior high school	737 (40.8)	350 (38.0)	387 (43.7)
College or higher	381 (21.1)	205 (22.3)	176 (19.9)
Missing	106 (5.9)	71 (7.7)	35 (4.0)

*GC represents gender contentedness; s.d., standard deviation; NTD, New Taiwan Dollar. GC1 refers to those who were consistently satisfied with their gender at both waves 1 and 9; GC2, those who were gender dissatisfied at wave 1 but satisfied at wave 9; GC3, those who were gender satisfied at wave 1 but dissatisfied at wave 9; GC4, those who were dissatisfied with their gender at both waves 1 and 9*.

**Table 2:** Comparison of delinquent behaviors, psychological well-being, self-esteem, and smoking/drinking for the different GC trajectory groups.

	**Delinquent behaviors**		**Psychological well-being (poor)**		**Self-esteem**		**Smoking/drinking**	
	**mean (s.d.)**		**mean (s.d.)**		**mean (s.d.)**		**mean (s.d.)**	
	**Wave 1**	**Wave 9**	**Wave 1**	**Wave 9**	**Wave 1**	**Wave 9**	**Wave 1**	**Wave 9**
**Male**								
GC trajectory groups								
GC1	5.39 (1.35)	5.63 (1.06)	22.22 (7.67)	23.35 (8.35)	24.28 (4.00)	24.50 (3.92)	1.10 (0.44)	1.75 (1.03)
GC2	5.90 (1.94)	5.58 (0.86)	27.87 (13.97)	23.88 (9.74)	20.82 (4.46)	23.08 (2.90)	1.20 (0.55)	1.92 (1.08)
GC3	5.36 (0.81)	6.4 (3.46)	18.00 (2.65)	25.06 (10.03)	24.68 (4.10)	24.71 (4.61)	1.05 (0.21)	1.77 (0.94)
GC4^a^	6.00 (1.00)	5.00 (–)	22.37 (7.96)	35.00 (–)	24.33 (2.08)	17.00 (–)	1.33 (0.58)	2.00 (–)
*F*	1.57	4.26^*^	5.31^**^	1.09	6.92^**^	2.36	1.01	0.25
Bonferroni test	–	a-c, b-c	a-b, b-c	–	a-b, b-c	–	–	–
**Female**								
GC trajectory groups								
GC1	5.21 (0.67)	5.30 (0.67)	23.61 (6.91)	24.81 (7.54)	24.03 (3.89)	23.90 (3.88)	1.05 (0.27)	1.34 (0.65)
GC2	5.44 (1.05)	5.55 (1.00)	27.17 (9.82)	26.24 (7.42)	23.23 (4.28)	23.11 (4.20)	1.07 (0.26)	1.32 (0.52)
GC3	5.17 (0.38)	5.19 (0.52)	23.60 (7.12)	25.36 (6.21)	23.38 (3.10)	22.31 (3.80)	1.02 (0.15)	1.27 (0.70)
GC4	5.27 (0.46)	5.86 (1.03)	34.00 (13.08)	29.71 (6.06)	20.71 (4.68)	20.79 (4.77)	1.07 (0.26)	2.07 (1.05)
*F*	3.35^*^	5.9^**^	15.3^**^	2.78^*^	4.58^*^	5.22^**^	0.44	6.04^**^
Bonferroni test	a-b	a-b, a-d, c-d	a-b, a-d, b-d, c-d	–	a-d	a-d	–	a-d, b-d, c-d

* *p < 0.05*,

** *p < 0.01. GC represents gender contentedness; s.d., standard deviation. GC1 represented those who were consistently satisfied with their gender at both waves 1 and 9; GC2, those who were dissatisfied with their gender at wave 1 but satisfied at wave 9; GC3, those who were satisfied with their gender at wave 1 but dissatisfied at wave 9. GC4, those who were consistently dissatisfied with their gender at both waves 1 and 9*.

a *GC4 group was not included in the multiple comparisons because of its small size (N = 3)*.

In the multiple linear regression analyses, Model 1, which considered the sole effect of the GC trajectory, showed that boys in GC2 were at increased risk for poor psychological well-being [β = 6.165, (95% CI = 3.148, 9.181)] and low self-esteem [β = −3.780, (−5.418 to −2.141)] at wave 1 (Table 3). Similarly, boys in GC3+4 had a higher likelihood of engaging in delinquent behavior [β = 0.635 (0.237, 1.033)] and of having poor psychological well-being [β = 3.125, (0.074, 6.175)]. In Model 2, which took the effect of PT and GC into consideration, the estimated effects of the GC trajectory on the outcome variables was not changed to any great degree, while earlier PT was positively associated with self-esteem at wave 1 [β = 0.370, (0.078, 0.662)] but negatively at wave 9 [β = −0.429, (−0.742 to −0.115].

**Table 3:** Multiple linear regression analysis of the association between gender contentedness, pubertal timing, and psychological and behavioral outcomes in males.

	**Delinquent behavior**		**Psychological well-being (poor)**		**Self-esteem**		**Smoking/drinking**	
	**β** **(95% CI)**		**β** **(95% CI)**		**β** **(95% CI)**		**β** **(95% CI)**	
	**Wave 1**	**Wave 9**	**Wave 1**	**Wave 9**	**Wave 1**	**Wave 9**	**Wave 1**	**Wave 9**
**Model 1**								
GC trajectory groups								
GC1	–	–	–	–	–	–	–	–
GC2	0.030 (−2.658 to 2.717)	−0.210 (−0.680 to 0.261)	6.165^**^ (3.148, 9.181)	−2.387 (−6.063 to 1.289)	−3.780^**^ (−5.418 to −2.141)	−0.468 (−2.310 to 1.375)	0.121(-.054, 0.295)	0.052(−0.389 to 0.493)
GC3 + 4	−0.634 (−2.736 to 1.469)	0.635^*^ (0.237, 1.033)	−0.691 (−3.225 to 1.843)	3.125^*^ (0.074, 6.175)	0.059 (−1.249 to 1.367)	−0.090 (−1.551 to 1.371)	−0.049 (−0.193 to 0.096)	−0.005(-.377, 0.367)
**Model 2**								
GC trajectory groups								
GC1	–	–	–	–	–	–	–	–
GC2	0.001 (−2.69, 2.71)	−0.210 (−0.682 to 0.263)	7.38^**^ (4.22, 10.54)	−2.510 (−6.189, 1.169)	−0.399^**^ (−5.73 to −2.24)	−0.461 (−2.292 to 1.370)	0.144 (−0.043 to 0.330)	0.045 (−0.398 to 0.488)
GC3 + 4	−0.061 (−2.72 to 1.51)	0.624^*^ (0.224, 1.024)	−0.605 (−3.11 to 1.90)	3.040 (−0.016 to 6.096)	0.009 (−1.22 to 1.40)	−0.237 (−1.692 to 1.218)	−0.049 (−0.196 to 0.099)	−0.001 (−0.375 to 0.374)
Pubertal timing	0.139 (−0.329 to 0.606)	−0.026 (−0.111 to 0.059)	0.553 (0.194, 0.911)	−0.325 (−0.981 to 0.332)	0.370^*^ (0.078, 0.662)	−0.429^*^ (−0.742 to −0.115)	0.027 (−0.006 to 0.059)	0.026 (−0.054 to 0.106)
**Model 3**								
GC trajectory groups								
GC1	–	–	–	–	–	–	–	–
GC2	−0.003 (−2.76 to 2.71)	−0.203 (−0.682 to 0.276)	7.141^**^ (3.943, 10.338)	−3.027 (−6.731 to 0.677)	−2.748^**^ (−5.777 to −0.282)	−0.446 (−2.281 to 1.389)	0.116 (−0.075 to 0.306)	0.018 (−0.430 to 0.466)
GC3 + 4	−0.631 (−2.815, 1.553)	0.605^*^ (0.181, 1.029)	−0.482 (−3.075, 2.111)	2.041 (−1.180, 5.261)	0.120 (−1.230 to 1.469)	−0.466 (−2.007 to 1.076)	−0.063 (−0.215 to 0.089)	−0.025 (−0.421 to 0.372)
Pubertal timing	0.136 (−0.352 to 0.623)	−0.023 (−0.111 to 0.066)	0.283 (−0.293 to 0.858)	−0.327 (−1.005 to 0.351)	0.341^*^ (0.037, 0.645)	−0.325^*^ (−0.718 to −0.069)	0.022 (−0.012 to 0.056)	0.022 (−0.061 to 0.105)
**Interaction effects**								
GC2 × PT	0.178 (−2.229 to 2.584)	−0.038 (−0.479 to 0.403)	1.376 (−1.437 to 4.188)	2.997 (−0.351 to 6.345)	0.653 (−0.911 to 2.217)	−0.258 (−1.973 1.457)	0.174^**^ (0.006 to 0.341)	0.170 (−0.244 to 0.583)
GC3 + 4 × PT	−0.117 (−2.523 to 2.289)	−0.066 (−0.546 to 0.414)	0.550 (−2.272 to 3.372)	−3.425 (−7.071 to 0.221)	0.142 (−1.344 to 1.628)	−0.475 (−2.535 to 0.953)	−0.063 (−0.230 to 0.105)	−0.077 (−0.526 to 0.372)

* *p < 0.05*,

** *p < 0.01*.

*GC represents gender contentedness; Pt, pubertal timing; CI, confidence interval. GC1 represented those who were consistently satisfied with their gender at both waves 1 and 9; GC2, those who were dissatisfied with their gender at wave 1 but satisfied at wave 9; GC3 + 4, those who were dissatisfied with their gender at wave 9. All regression analyses used GC1 as the reference group, and when analyzing the outcome variable at wave 9, we also included the value at wave 1 in the model to adjust for its time–dependent effect*.

As to the females in Model 1, those in GC2 were at increased risk for engaging in more delinquent behaviors [β = 0.262, (0.106, 0.418)], poorer psychological well-being [β = 3.949, (2.275, 5.622)], and lower self-esteem [β = −1.117, (−1.984 to −0.249)] at wave 1 and more delinquent behaviors [β = 0.259, (0.076, 0.441)] at wave 9 (Table 4). Those in GC3 + 4 had a higher likelihood of having low self-esteem at both waves 1 [β = −1.179, (−2.326 to −0.031)] and 9 [β = −1.770, (−3.003 to −0.537)]. In Model 2, earlier PT was found be associated with poorer psychological well-being and lower self-esteem at wave 1, while those in GC3 + 4 had an augmented risk for smoking and drinking [β = 0.223, (0.005, 0.441)] at wave 9.

**Table 4:** Multiple linear regression analysis of the association between gender contentedness, pubertal timing, and psychological and behavioral outcomes in females.

	**Delinquent behavior**		**Psychological well-being (poor)**		**Self–esteem**		**Smoking/drinking**	
	**β** **(95% CI)**		**β** **(95% CI)**		**β** **(95% CI)**		**β** **(95% CI)**	
	**Wave 1**	**Wave 9**	**Wave 1**	**Wave 9**	**Wave 1**	**Wave 9**	**Wave 1**	**Wave 9**
**Model 1**								
GC trajectory groups								
GC1	–	–	–	–	–	–	–	–
GC2	0.262^**^ (0.106, 0.418)	0.259^*^ (0.076, 0.441)	3.949^**^ (2.275, 5.622)	−0.246 (−2.086 to 1.595)	−1.117^*^ (−1.984 to −0.249)	−0.021 (−1.005 to 0.963)	0.006 (−0.051 to 0.063)	−0.019 (−0.185 to 0.147)
GC3 + 4	0.000 (−0.209 to 0.209)	0.048 (−0.181 to 0.276)	2.244 (0.009, 4.479)	1.192 (−1.106 to 3.490)	−1.179^*^ (−2.326 to −0.031)	−1.770^*^ (−3.003 to −0.537)	−0.006 (−0.082 to 0.070)	0.192 (−0.018 to 0.402)
**Model 2**								
GC trajectory groups								
GC1	–	–	–	–	–	–	–	–
GC2	0.267^**^ (0.103, 0.430)	0.281^*^ (0.089, 0.472)	3.603^**^ (1.809, 5.396)	−0.086 (−2.016 to 1.845)	−0.963^*^ (−1.876 to −0.050)	0.020 (−0.998 to 1.038)	0.011 (−0.050 to 0.071)	−0.016 (−0.186 to 0.155)
GC3 + 4	−0.001 (−0.219 to 0.221)	0.084 (−0.160 to 0.327)	2.829^*^ (0.387, 5.270)	0.862 (−1.599 to 3.323)	−1.076 (−2.304 to 0.153)	−1.808^*^ (−3.105 to −0.511)	−0.017 (−0.098 to 0.064)	0.223^*^ (0.005, 0.441)
Pubertal timing	0.031 (−0.021 to 0.082)	−0.012 (−0.072 to 0.048)	1.224^**^ (0.654, 1.794)	0.145 (−0.464 to 0.753)	−0.285^*^ (−0.569 to 0.000)	−0.0218 (−0.533 to 0.098)	0.017 (−0.002 to 0.036)	0.052 (−0.002 to 0.105)
**Model 3**								
GC trajectory groups								
GC1	–	–	–	–	–	–	–	–
GC2	0.250^*^ (0.082, 0.417)	0.303^*^ (0.103, 0.503)	3.554^**^ (1.720, 5.387)	−0.150 (−2.172 to 1.872)	−0.097^*^ (−2.061 to −0.192)	0.096 (−0.986 to 1.177)	0.018 (−0.044 to 0.079)	0.023 (−0.156 to 0.201)
GC3 + 4	0.003 (−0.219 to 0.224)	0.062 (−0.183 to 0.306)	2.868^*^ (0.413, 5.322)	0.877 (−1.598 to 3.351)	−1.172 (−2.405 to 0.062)	−1.830^*^ (−3.137 to −0.523)	−0.017 (−0.098 to 0.065)	0.213 (−0.006 to 0.432)
Pubertal timing	0.020 (−0.037 to 0.077)	0.012 (−0.054 to 0.078)	1.163^**^ (0.529, 1.797)	0.167 (−0.564 to 0.777)	−0.305 (−0.620 to 0.010)	−0.177 (−0.524 to 0.170)	0.021^*^ (0.000, 0.042)	0.059^*^ (0.013, 0.131)
**Interaction effects**								
GC2 × PT	0.081 (−0.081 to 0.243)	−0.091 (−0.290 to 0.109)	0.259 (−1.520 to 2.037)	0.229 (−1.758 to 2.216)	0.668 (−0.240 to 1.575)	−0.244 (−1.322 to 0.835)	−0.032 (−0.092 to 0.028)	−0.132 (−0.310 to 0.046)
GC3 + 4 × PT	0.021 (−0.175 to 0.217)	−0.203 (−0.435 to 0.029)	0.417 (−1.751 to 2.585)	0.200 (−2.139 to 2.538)	−0.708 (−1.786 to 0.371)	−0.231 (−1.449 to 0.988)	−0.003 (−0.075 to 0.069)	−0.105 (−0.312 to 0.102)

* *p < 0.05*,

** *p < 0.01*.

*GC represents gender contentedness; PT, pubertal timing; CI, confidence interval. GC1 represented those who were consistently satisfied with their gender at both waves 1 and 9; GC2, those who were dissatisfied with their gender at wave 1 but satisfied at wave 9; GC3+4, those who were dissatisfied with their gender at wave 9. All regression analyses used GC1 as the reference group, and when analyzing the outcome variable at wave 9, we also included the value at wave 1 in the model to adjust for its time-dependent effect*.

In Model 3, we added an interaction term between GC and PT. In males, we only found a significant interaction term between the GC trajectory and PT associated with smoking and drinking at wave 1. However, no significance was found for the interaction term between the GC trajectory and PT in females.

## Discussion

This is the first study, to the best of our knowledge, to survey gender issues among Taiwanese adolescents and track them down across development. Using GC as a proxy of gender identity, the results showed that the rate of gender discontentment was 10.7 and 5.7% in adolescence and young adulthood, which is comparable to but slightly higher than the estimated rate of gender dysphoria reported previously in incoming university students (20). Our study clearly demonstrated longitudinal GC changes over adolescence among a representative youth cohort. We found that ~10% of the males and 20% of the females changed their GC at some point over the entire adolescence, which supported the cognitive development theory of gender identity (10) and the use of GC as a reliable and alternative indicator of gender identity (7). Although gender identity is usually in agreement with the assigned gender and seems to be fairly fixed from early childhood (30), it sometimes changes over time corresponding to the development of conceptual abilities (10). When gender identity does not align with biological gender, one may develop gender dysphoria or gender discomfort beyond simply feeling dissatisfied with gender (31). Contemporary feelings of gender discontentedness or even discomfort may be influenced by some contextual factors. Previous research has summarized three potential contributing mechanisms to an increase or decrease in gender discomfort and gender identification in adolescence: (1) physical puberty, (2) the social context regarding natal sex, and (3) the discovery of sexuality (32). For example, physical puberty brought about by sex hormones may intensify gender dysphoria, if it existed earlier (32, 33). Social values and contextual changes may affect explicit expression of gender identity, especially in gender non-conforming individuals, who face stress from family, school, and even themselves (32). Also, anticipated body image and seeking the experience of falling in love or a sexual relationship may shift the process of self-identification (32).

We further examined the time-varying relationship between GC and psychosocial health outcomes in adolescence and young adulthood. Aligned with previous research (34), it was found that gender discontentment was associated with poor psychological well-being and low self-esteem both in males and females and more delinquent behavior in females at baseline in adolescence. Only the relationship with delinquent behavior remained significant in the females in the GC2 group, who outgrew gender discontentment and became gender-satisfied. As such, we argue that adolescent psychosocial development is subject to perceived GC and that gender discontentment is indeed temporarily associated with internalizing and externalizing maladaptation. Most maladaptive problems can be outgrown over adolescence and young adulthood. It is noteworthy that some delinquent behaviors may carry on if they are formed earlier in adolescence, particularly in the case of females. Given that our GC measurements were available at two time points, it is not allowed to further characterize the pattern of GC changes at the passage of entire developmental course. That is, whether GC oscillates over time and how this oscillation, if extant, implies in adolescent health and development require more research to explore.

Consistent with previous research on the link between early PT and several emotional and behavioral problems (35), our results showed early PT to be associated with depressive symptoms and low self-esteem at baseline in females, but these associations were not shown to be significant in young adulthood. Contrarily, in their male counterparts, earlier PT was associated with higher self-esteem in adolescence but lower self-esteem in young adulthood. This reverse association seen in boys' different life stages may be related to the advantageous physical growth brought about by early sexual maturation at puberty but reduced adult height due to early growth plate closure in young adulthood (36). Meanwhile, we found that after controlling PT, the estimated effects of GC remained significantly important, again reflecting the critical role of GC in determining adolescent psychosocial development. Further, we researched the interaction effect between GC and PT in Model 3. An interaction effect appeared significant in the relationship with smoking and drinking in males. It is tentatively concluded that gender discontent and off-pubertal timing are likely to interact and to have an adverse effect on adolescent smoking and drinking behavior. Aligned with previous research findings that teenage smoking and drinking habits may result from social anxiety (37), our results can be better explained by an anxious state that was created by physical signs appearing during pubertal progression and self-perceived gender non-compatibility (38). However, the association was not persistent into young adulthood. Some studies supported that those who vented their negative feelings by alcohol and cigarette consumption in adolescence would have a lower risk for using these two substances in early adulthood.

This study has some limitations. First, the survey on GC was dichotomous, using a single question. Only one question measuring GC is a quite limited measure and prone to misunderstanding. In addition, it was different from Egan and Perry's concept of gender contentedness that asked how content one is with one's socially prescribed gender role. Even though some teenagers are definably gender-non-conforming, they may have different levels of GC and different understanding of this question. Also more sophisticated conceptual constructs may be needed to obtain a global measurement of gender identity, such as gender role, social attributes, and sexual orientation. Second, the health outcome variables were self-reported. Under this circumstance, fear of repercussions may have prevented participants who engaged in delinquent behavior or substance use from reporting this behavior accurately. In particular, some illegal behaviors, such as illicit drug use in Taiwan, are very likely subject to underreporting in a questionnaire-based survey although this reporting bias may be partially compensated for by the anonymity of questionnaire. Third, data were collected between 2000 and 2009. They may not reflect current rapid increase and changes in diverse gender expressions among adolescents. Nonetheless, it has been argued that analyzing archived data could inspire new ideas or refine existing literature (39, 40). Readers should be careful about this time gap, while further study with updated dataset may contribute to verifying our findings. Last, other potential confounders and potentially relevant cultural factors related to the outcomes, with the exception of parental education and family incomes, were not examined in our study due to a lack of their availability in a secondary dataset analysis. Future studies including comprehensive confounding variables may be needed to corroborate our results.

## Conclusion

Our results described the longitudinal GC trajectory over adolescence and young adulthood in a representative youth cohort, where GC can be a reliable and non-judgmental indicator of gender identity for the purpose of population survey. Approximately 10% of males and 20% of females may change their GC perceptions at some point in time. Gender discontentment may be associated with adverse psychological and behavioral outcomes in adolescence, and some delinquent behaviors may persist into young adulthood. Based on these results, we propose some clinical and public health implications for adolescent health care. First, although gender identity is usually fixed before adolescence, healthcare providers should assess individual perceptions of gender when consulting adolescents for psychological and behavioral issues. The associations between the GC trajectory and psychological and behavioral outcomes may reflect the underlying social anxiety imposed on gender non-conforming individuals. Adverse effects of the GC trajectory on some externalizing behaviors, such as substance use and delinquent behavior, can be persistent and thus should be scrutinized until young adulthood. Preventive and interventional measures should be promptly initiated at early adolescence if behavioral problems are taking place. Second, gender differences and interaction effects with PT were observed in the stratified analysis. Thus, individual counseling should be tailored according to gender and pace of pubertal progression to help adolescents better understand their internal gender identities and enable them to soundly navigate toward their affirmed gender.

## Data Availability Statement

Publicly available datasets were analyzed in this study. This data can be found here: https://srda.sinica.edu.tw/browsingbydatatype_result.php?category=surveymethod&type=2&csid=1.

## Ethics Statement

The studies involving human participants were reviewed and approved by National Cheng Kung University Hospital. Written informed consent to participate in this study was provided by the participants' legal guardian/next of kin.

## Author Contributions

J-HK and M-CT conceived the study and drafted the manuscript. J-HK, RA, and LC conducted the analysis, while CS, M-CT, and C-YL supervised the analysis. Y-CL and Y-PH contributed the development of study and critically reviewed the manuscript. All authors read and approved the final version of the manuscript.

## Funding

This work was supported by a research grant awarded to M-CT from the Ministry of Science and Technology, Taiwan (108-2629-B-006−002) and by a Summer Research Project Grant awarded to J-HK from the College of Medicine at National Cheng Kung University (NCKUMCS2019014).

## Conflict of Interest

The authors declare that the research was conducted in the absence of any commercial or financial relationships that could be construed as a potential conflict of interest.

## Publisher's Note

All claims expressed in this article are solely those of the authors and do not necessarily represent those of their affiliated organizations, or those of the publisher, the editors and the reviewers. Any product that may be evaluated in this article, or claim that may be made by its manufacturer, is not guaranteed or endorsed by the publisher.

## Abbreviations

CI: confidence interval
GC: gender contentedness
NTD: New Taiwanese Dollar
PT: pubertal timing
TYP: Taiwan Youth Project.
